# Radiomics Features Predict CIC Mutation Status in Lower Grade Glioma

**DOI:** 10.3389/fonc.2020.00937

**Published:** 2020-06-26

**Authors:** Luyuan Zhang, Felipe Giuste, Juan C. Vizcarra, Xuejun Li, David Gutman

**Affiliations:** ^1^Department of Neurosurgery, Xiangya Hospital, Central South University, Changsha, China; ^2^Department of Neurosurgery, First Affiliated Hospital, School of Medicine, Zhejiang University, Hangzhou, China; ^3^Department of Biomedical Engineering of the Georgia Institute of Technology, Emory University, Atlanta, GA, United States; ^4^Department of Neurology, Emory University, Atlanta, GA, United States

**Keywords:** glioma, radiomics, MRI, CIC, prediction

## Abstract

MRI in combination with genomic markers are critical in the management of gliomas. Radiomics and radiogenomics analysis facilitate the quantitative assessment of tumor properties which can be used to model both molecular subtype and predict disease progression. In this work, we report on the Drosophila gene capicua (CIC) mutation biomarker effects alongside radiomics features on the predictive ability of CIC mutation status in lower-grade gliomas (LGG). Genomic data of lower grade glioma (LGG) patients from The Cancer Genome Atlas (TCGA) (*n* = 509) and corresponding MR images from TCIA (*n* = 120) were utilized. Following tumor segmentation, radiomics features were extracted from T1, T2, T2 Flair, and T1 contrast enhanced (CE) images. Lasso feature reduction was used to obtain the most important MR image features and then logistic regression used to predict CIC mutation status. In our study, CIC mutation rarely occurred in Astrocytoma but has a high probability of occurrence in Oligodendroglioma. The presence of CIC mutation was found to be associated with better survival of glioma patients (*p* < 1e−4, HR: 0.2445), even with co-occurrence of IDH mutation and 1p/19q co-deletion (*p* = 0.0362, HR: 0.3674). An eleven-feature model achieved glioma prediction accuracy of 94.2% (95% CI, 94.03–94.38%), a six-feature model achieved oligodendroglioma prediction accuracy of 92.3% (95% CI, 91.70–92.92%). MR imaging and its derived image of gliomas with CIC mutation appears more complex and non-uniform but are associated with lower malignancy. Our study identified CIC as a potential prognostic factor in glioma which has close associations with survival. MRI radiomic features could predict CIC mutation, and reflect less malignant manifestations such as milder necrosis and larger tumor volume in MRI and its derived images that could help clinical judgment.

## Introduction

Glioma is the most common primary tumor in the adult central nervous system (CNS). High-grade gliomas (grade IV) have poor median survival [~14 months (1)] compared with grade II and III (2). In 2016, the World Health Organization (3) updated its glioma classification scheme to incorporate genomic information including IDH (Isocitrate dehydrogenase) mutation and 1p/19q codeletion (4). In addition to facilitating the diagnosis of gliomas, genomic information is also used in guiding the extent of surgical tumor resection and therapeutic strategy. In patients with IDH mutation, gross total resection (GTR) has been found to result in longer survival times compared to non-GTR (5, 6). Although confirming the genetic status of glioma is instructive for surgery and post-surgical treatment, it is still subject to methodological limitations. Neurosurgical biopsies during craniotomy are the current standard used to obtain genomic information about glioma. However, a single biopsy is unlikely to represent the full set of mutations present in the cancer due to high tumor genomic and histological heterogeneity (7–9). Therefore, there is a need to develop a method that can reflect the global characteristics of gliomas which is robust to regional variation and provides clinically actionable conclusions.

The homolog of the Drosophila gene capicua (CIC) gene is a member of the high mobility group (HMG)-box superfamily of transcriptional repressors on chromosome 19q. The role of CIC mutations in human disease is still unclear. It has been reported that CIC mutation promotes glioma cell proliferation, differentiation, and aggression and results in a poor outcome (10–12). However, Jiao et al. (13) found that patients with IDH mutations combined with either 1p19q loss, FUBP1 mutations, or CIC mutations will have longer overall survival than patients with IDH mutations combined with ATRX mutation. However, because CIC mutation is closely related to IDH mutation and 1p/19q co-deletion, whether CIC mutation is an independent prognostic factor remains to be clarified. In addition, CIC mutation tends to occur in oligodendroglioma but not in the astrocytoma (14, 15). But associated studies are mainly based on the 2007 WHO classification; whether these findings will remain when employing the latest 2016 WHO classification still needs to be explored.

Because of the heterogeneity of gliomas, genomic and histological data obtained from biopsies can fail at representing the entire glioma heterogeneity. Magnetic resonance imaging (MRI) provides a possibility to break this limitation, since information about the entire glioma can be obtained. VASARI (Visually AcceSAble Rembrandt Images) MRI features (16) and radiomics features (17) are two common methods to extract features from MR images. Radiomics is a process that converts digital medical images into mineable high-dimension data (18). It provides high-dimensional quantitative information and comprehensive information regarding tumor heterogeneity (18) that may fail to be appreciated by the naked eye of radiologists. Radiogenomics is an emerging field that explores the associations between radiomics and genomics (19). IDH mutation and 1p/19q codeletion have been predicted accurately by radiomics features (20–23), but there has been no reports using radiomics features to predict CIC mutation. Another obstacle of radiomics features is that they are difficult to understand and cannot be related to tumor physiological changes (24).

In this study we aim to identify the value of CIC mutations in gliomas by analyzing the relationship between CIC mutations and the clinical characteristics, key molecular markers, and patient survival. Then, by extracting radiomics features from lower-grade glioma MRI, a robust CIC mutation prediction model is established. The relationship between key features and glioma structural changes in MRI is analyzed to explore the possible physiological changes of gliomas behind structural changes.

## Materials and Methods

### Data Sources

A total of 516 lower-grade glioma (LGG) patients' genomic data and clinical data were downloaded from the TCGA data portal [https://portal.gdc.cancer.gov/]. Among these 516 TCGA patients, 199 patients have MR images stored in the Cancer Imaging Archive (TCIA) (25). Additional genomic and clinical metadata of TCGA was obtained through cBioPortal (26, 27). In addition, the genomics dataset of glioblastoma was also obtained from cBioPortal. All TCGA related data were previously anonymized and are publicly available.

### Genomics Data

All genomic data were downloaded from the TCGA dataset. Single nucleotide polymorphism (SNP) data was used to identify gene mutations, including CIC and IDH. Missense, frameshift, and nonsense mutations were included in the definition. Copy number variation (CNV) data was used to identify 1p/19q co-deletion status. A segment mean value < −0.2 was considered as deletion in the corresponding region (28). Because TCGA CNV probes didn't cover the whole chromosome, 1p/19q codeletion status was derived using copy number data as shown in (29).

### Histological Type

There are two different WHO CNS tumor classifications, namely from 2007 and 2016. The 2007 classification used in the TCGA defines the histological types as Astrocytoma, Oligodendroglioma, and Oligoastrocytoma. The 2016 classification incorporated molecular biomarkers in their classification scheme, mainly IDH mutation and 1p/19q co-deletion. Oligodendroglioma is defined as Glioma with IDH mutation and 1p/19q co-deletion, and Diffuse Astrocytoma is defined as glioma with IDH mutation but without 1p/19q co-deletion or IDH wild-type (3) (Figure S1).

### Image Pre-processing

Quality control (QC) was done manually by reviewing images on a local instance of the Digital Slide Archive (DSA) (30), which allows the rapid review of DICOM files. MRIcron (31) was then used to convert all images from DICOM format to NIFTI format for subsequent analysis.

### Image Masking

The FSL image viewer (FSLeyes 0.10.1) (32) was used to draw regions of interest (ROIs) slice-by-slice. A total of 120 T1-weighted (T1W), T1 contrast-enhanced (T1CE), and T2-weighted (T2W) image ROI masks, and FLAIR image ROI masks were generated. All radiomic features were extracted using the T1W image ROI mask.

### Image Processing Pipeline

**Figure 1:**
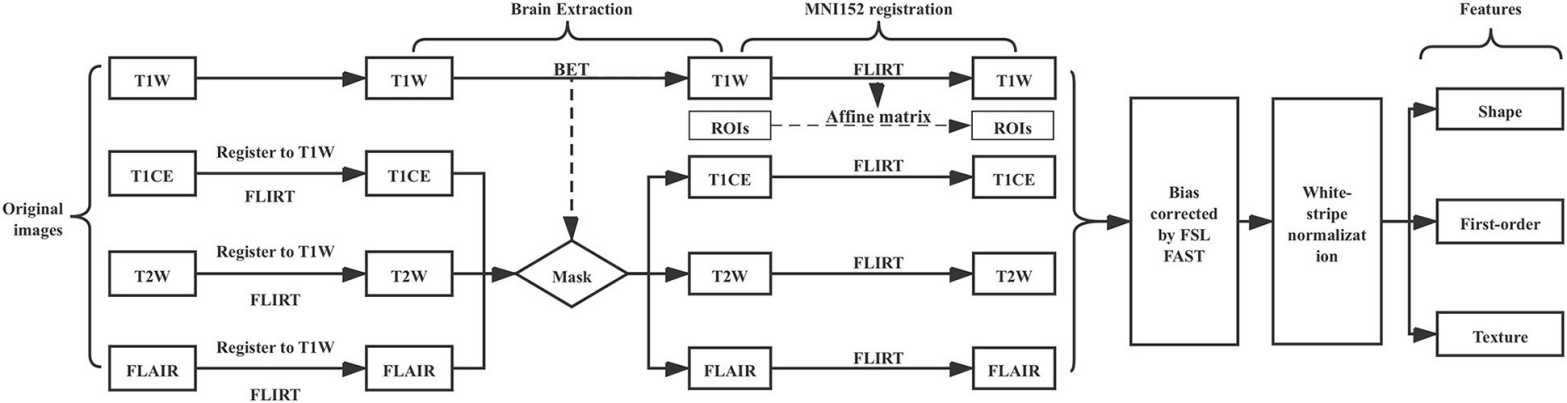
MRI preprocessing pipeline.

The image processing pipeline is illustrated in (Figure 1). First, we used the FSL Brain Extraction Tool (BET) to remove the skull, eyes, and other non-brain tissue within T1W images (33). We found that the quality of lower-grade glioma images from the TCIA is variable, oftentimes resulting in poor brain extraction using BET. To address this limitation, we manually corrected the BET extraction results to be consistent between images. This approach allowed us to obtain good quality brain tissue masks while speeding up the process in comparison to completely manual brain mask delineation. We used the T1W brain region as a mask to get T1CE, T2, and FLAIR images' brain tissue after we registered T1CE, T2W, and FLAIR to T1W images. FSL FLIRT was used for image registration. In order to make all patients' images comparable, we registered all images and masks to the 1 mm MNI152 atlas. Registered images were bias corrected by FSL FAST. White-stripe normalization (34) was conducted to normalize image intensities.

### Feature Extraction

Radiomics features were extracted using the Python package PyRadiomics V2.0.0 (35). PyRadiomics can perform various transformations on the original input image prior to extracting features. The transformations we used include: Original, Wavelet, Square, Square Root, Logarithm, Exponential, Gradient, Local Binary Pattern 2D (2D-LBP), and Local Binary Pattern 3D (3D-LBP). After image transformations, 105 radiomics features can be extracted from each transformed image using PyRadiomics, these features are summarized in Table S1. In 3D-LBP images, a rotational invariant operator using spherical harmonics was utilized. Three different radii for the spherical harmonics were used, with radius similar to those used in 2D-LBP images, resulting in three different 3D-LBP images. The information stored in the transformed images of different radii in 3D-LBP is different. In wavelet transformed images, each dimension in the 3D image was divided into high frequency components (H) and low frequency components (L). Combining the H and L of three different dimensions of the 3D image can produce eight different combinations: LLL, LLH, LHL, LHH, HLH, HHL, HLL, HHH.

### Feature Selection

LassoCV in the scikit-learn Python package was used for radiomics feature selection (36). It combines cross-validation (CV) and Lasso regression. The advantage of LassoCV is that it does not need to manually set regularization coefficient (λ). It can try the default series of λ through CV iteration, and then automatically select the best model (Figure S2). In LassoCV, to avoid selection bias due to the low proportion of CIC mutations, we used stratified sampling. Both 10-fold CV and 5-fold CV are common (37) but limited by the number of CIC mutation samples, the variance of the 10-fold CV will be great (38), so we choose 5-fold CV. “StratifiedKFold” in the scikit-learn Python package was used. Before the CV splitter splits the samples, all samples are shuffled.

Because of 5-fold CV and data shuffle, only 80% of the total samples were used to train the Lasso model, and these samples should be different each time the Lasso model is built (Figure S2). In addition, for some highly relevant features, Lasso will randomly select one and exclude the others. This results in the features selected by LassoCV not being the same every time. But the probability of important features being selected is always large, so we repeated LassoCV 100 times (Figure S3). The selected features and its coefficient each time were recorded. The features whose sum of the coefficients unequal to zero are included. Features are sorted according to the number of times selected, and the top $\sqrt[{2}]{n}$ (*n*: sample size) (39) features are selected, so, 11($\sqrt[{2}]{120}$) and 6($\sqrt[{2}]{35}$) radiomics features were used to predict the CIC mutation in glioma and oligodendroglioma, respectively.

In order to detect the collinearity between the radiomics features, we performed a Pearson product moment correlation coefficient analysis between the radiomics features, then clustered the correlation coefficients between the features, and then used the clustermap to visualize.

### Texture-Based CIC Prediction

A logistic regression model, defined by the function below, was created in Python utilizing the SciKit-Learn package:

$$\begin{array}{l} h_{\theta }\left(\chi \right)=\frac{1}{1+e^{-z}} \end{array}$$

In the model, *h*_θ_(χ) is the estimated probability of CIC mutation status. CIC mutation presence is defined as one, and absence is defined as zero. *z* represents ordinary linear regression:

$$\begin{array}{l} z=\theta_{0}+\theta_{1}\chi_{1}+\theta_{2}\chi_{2}+\theta_{3}\chi_{3}...+\theta_{n}\chi_{n} \end{array}$$

*z* is the dependent variable. *n* represents the number of features. (χ_1_,χ_2_,χ_3_...χ_*n*_) is independent variables. (θ_1_,θ_2_,θ_3_...θ_*n*_) is features' partial regression coefficient. θ_0_is the intercept of the linear model.

Because the CIC mutation in our dataset is unbalanced, the weight of two classes are corrected by: n_samples / [n_classes ^*^ n_label (CIC mutant or CIC wild-type)]. All features were z-scored before being placed in the model. Because the unit differences between features are eliminated, the coefficients of each feature in the prediction model represent the importance of the feature in the model.

### Statistics

Univariate Cox regression was used to find associations between gene mutation and survival. To analyze the classification, clinical characteristics, and other known molecular markers of gliomas and the relationship between CIC mutations, we used the two-sided Chi-square test. To analyze the prognostic value of CIC mutations as molecular markers, we used Log-rank test, Kaplan-Meier survival analysis, and multivariate COX regression analysis. We used the Log-rank test to analyze the relationship between IDH mutation, 1p/19q co-deletion and CIC mutation and overall survival, and Kaplan-Meier survival analysis curve to visualize. To identify whether the CIC mutation is an independent prognostic factor, multivariate Cox tests were used, including age, gender, grade, histological type, IDH mutation, 1p/19q codeletion, and FUBP1 mutation as covariates. The differences were considered significant if the *p*-value was < 0.05. The image dataset was stratified random sampling into training and testing sets (80% train, 20% test). Training set was used to train the logistic model and the test set was used to test model performance. Because of the stratified random split of the dataset, there will be differences between the training set and the test set each time, resulting in different trained logistic regression models and prediction results, so we repeat the above process 1,000 times (Figure S4). Then we will obtain 1,000 logistic regression models trained by different training sets and the corresponding prediction results. So we sum coefficients of each feature of these 1,000 models as the importance of features. The mean AUC, prediction accuracy, sensitivity, and specificity of model were calculated for the testing set. Receiver operating characteristic (ROC) curve and Precision-recall (PR) curve analysis was conducted to evaluate the models. The coordinate points of the ROC curve and PR curve of 1,000 prediction models are averaged to obtain the average ROC curve and PR curve. The optimal cutoff value in the ROC curve and PR curve is the coordinate point closest to the upper left corner (0,1.0) and the upper right corner (1.0,1.0), respectively (40).

### Image Analysis

In order to evaluate the importance of radiomics features and its correlation with CIC mutations, we used the Mann-Whitney *U*-test to test features in the logistic model. *U*-test was performed on the features value of CIC mutation and wild-type samples. Significance was defined as *p* < 0.05. The radiomics features that are significant in *U*-test and ranked in top 1/3 by importance were used for further analysis. So 3 (11/3) radiomics features from the CIC mutation prediction model in glioma and 2 (6/3) radiomics features from the CIC mutation prediction model in oligodendroglioma will be selected. Images corresponding to the maximum and minimum values of the most significant features were selected. Because some radiomics features were extracted from transformed image, for these features, we show the transformed image but not the original input image. The probability estimates of each sample in the test set results of above mentioned 1,000 logistic regression models are summed, and then the average probability estimates of each sample are obtained. The samples with the largest and smallest average probability estimates are selected. The original T1W, T1CE, T2, and FLAIR images but not transformed images of these two samples were shown. Because the radiomics feature represents the information of the entire glioma, but the 3D image is not conducive to display, so we choose the one with the largest ROI area in the transverse plane slice to represent the entire glioma.

## Results

### Data Summary

**Figure 2:**
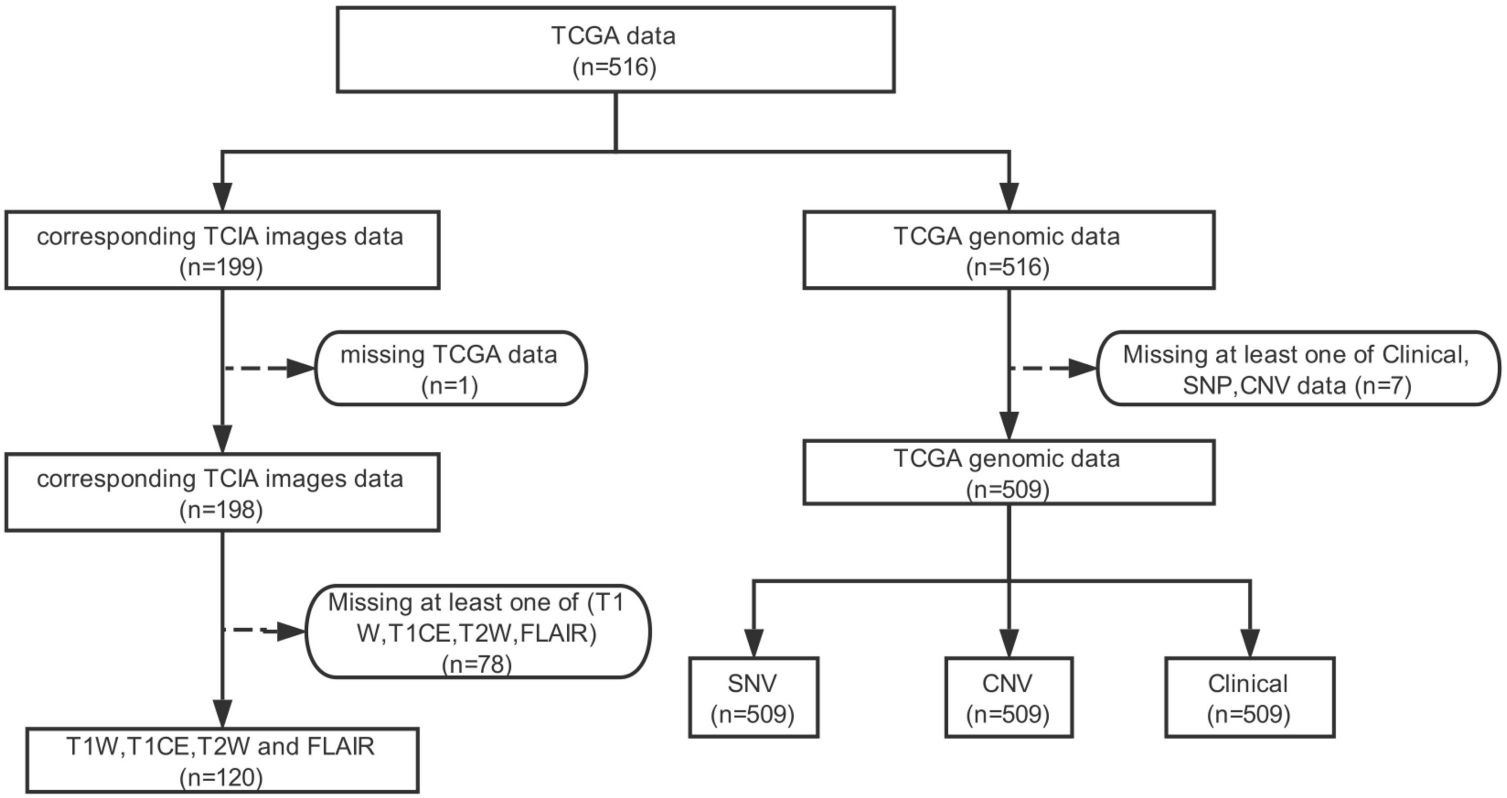
Data screening process.

Of the lower-grade gliomas cases downloaded from TCGA, 509 cases had CNV data, SNP data, and clinical data. This 509 cohort was used as our genomics dataset. One hundred ninety-nine MRI cases were downloaded from TCIA, 78 of which were removed due to the lack of at least one of T1W, T1CE, T2W, and FLAIR MRI, and one sample was removed due to the lack of corresponding genomic data in TCGA. A total of 120 cases remained and was used as the image dataset (Figure 2, Table S2). The two cohorts used in this work (TCGA LGG cohort and TCIA imaging cohort) did not differ significantly, with the exception of patient age (42.9 vs. 45.9; *p* = 0.0356) (Table S2).

### Identification of Mutation Frequency in the LGG Cohort

**Figure 3:**
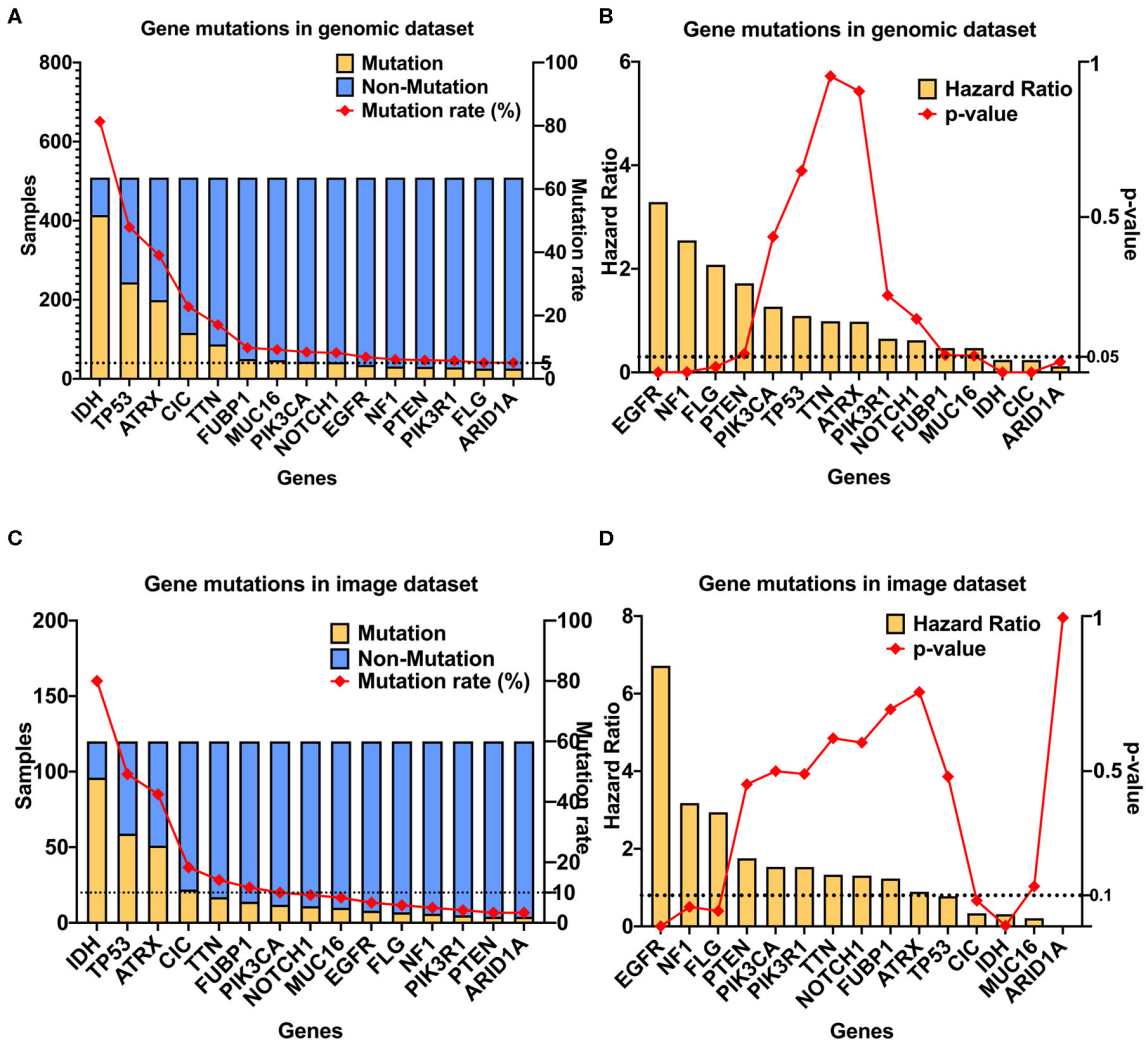
CIC mutation has a high occurrence rate and association with survival in LGG. **(A)** CIC mutation rate in gliomas. GBM, GBM dataset from cBioPortal (TCGA Cell 2103); LGG (509), TCGA LGG dataset; LGG (120), from TCIA. **(B)** Gene mutations in the TCGA dataset (*n* = 509), 15 gene mutation rates are >5%. **(C)** Gene mutations in the TCIA image dataset (*n* = 120), 12 gene mutation rates are >5%. **(D)** Multivariate Cox regression result of gene mutations in the genomic dataset, age was included as covariate. Only seven gene mutations are significant.

The cBioPortal was used to obtain information on multiple glioblastoma databases. Through the glioblastoma dataset in cBioPortal, we found that the incidence of CIC mutations in glioblastoma is low (0.3%). Our genomic dataset had a higher incidence of CIC mutations (22.8%), similar to the incidence of CIC mutations found in the imaging dataset (18.3%) (Figure S5). SNP was downloaded from the TCGA data portal for LGG patients, which identifies 14,014 unique SNPs. Among these, only 15 occurred in > 5% of patients (25 of 509 total patients) (Figure 3A). Six of these SNPs were significantly associated with overall patient survival. Among these six, EGFR (HR: 5.04, 95%CI: 3.16–8.02), NF1 (HR: 2.84, 95%CI: 1.64–4.91), and FLG (HR: 2.07, 95% CI: 1.13–3.78) mutation were associated with poor survival. IDH (HR: 0.17, 95% CI: 0.12–0.24), CIC (HR: 0.30, 95% CI: 0.17–0.55), and ARID1A (HR: 0.11, 95% CI: 0.02–0.80) mutation were found to improve survival (Figure 3B). We focused on genes that were present in at least 5% or more LGG cases for the genomic dataset. But on the imaging dataset, since the number of samples is smaller, we looked for genes present in at least 10% of the cases. Similarly, we also adjusted the *p*-value of survival regression to 0.1. In the imaging dataset, there are a total of seven gene mutations with an incidence rate >10%, namely IDH, TP53, ATRX, CIC, FUBP1, TTN, and PIK3CA mutation (Figure 3C). However, when considering only the samples in the imaging dataset, only IDH mutations (*p* = 0.0023, HR = 0.3166) and CIC mutations (*p* = 0.0831, HR = 0.3387) were significantly associated with survival (Figure 3D). Since IDH mutation has been the focus of previous studies (20, 41, 42), with high accuracy prediction results reported, we chose to focus on CIC mutation for our analysis. In conclusion, CIC mutation is the only molecular marker other than IDH mutation that satisfies the sufficiently large incidence, prognostic value, and conditions of radiomics prediction.

### Association Between CIC Mutations and Clinical Data

We analyzed the association between CIC mutations and clinical data in the genomics dataset. CIC mutations have close association with age, the probability of patients 50 years or older having CIC mutations is significantly greater than those <50 years old (*p* = 0.0140). The probability of grade 2 patients having CIC mutation is significantly greater than grade 3 patients (*p* = 0.0236). CIC mutation also have close association with the glioma location (*p* = 0.003967), the probability of CIC mutations in the temporal lobe is significantly lower than that in the frontal lobe (*p* = 0.0009) and the temporal lobe (*p* = 0.0792), but there is no significant difference between the frontal lobe and parietal lobe (*p* = 0.6411). CIC mutations are also related to clinical symptoms, the probability of samples without headache having CIC mutations is significantly greater than the probability of samples with headache (*p* = 0.0119), and there is a possible trend that the probability of CIC mutation in samples without visual change is greater than samples with visual changes (*p* = 0.0646), there is also a strong tendency that the probability of CIC mutation in samples without sensory change is greater than samples with sensory change (*p* = 0.0563) (Table 1).

**Table 1:** Associations between CIC mutation presence and clinical characteristics.

**Clinical characteristic**	**Subgroup**	**CIC mutation**	**CIC wild-type**	***P*-value^a^**
Age	<50 years old	68	278	0.01398
	≥50 years old	48	115	
Gender	Female	58	169	0.1828
	Male	58	224	
Grade	G2	67	180	0.02356
	G3	49	213	
KPS^b^	<90	17	75	0.3212
	≥90	48	155	
Cancer status	With tumor	53	216	0.1629
	Tumor free	45	133	
Tumor location	Frontal	81	217	0.003967
	Temporal	19	126	
	Parietal	11	35	
Laterality	Left	55	191	0.6083
	Right	61	190	
Seizures	Yes	73	224	0.3592
	No	37	140	
Headaches	Yes	27	142	0.0119
	No	76	215	
Mental status changes	Yes	21	92	0.2295
	No	82	259	
Visual changes	Yes	9	56	0.06461
	No	94	294	
Sensory changes	Yes	10	62	0.05633
	No	91	286	
Motor movement changes	Yes	29	81	0.304
	No	74	268	
First symptom	Seizures	57	188	0.5352
	Headaches	18	87	
	Mental status changes	8	31	
	Visual changes	3	9	
	Sensory changes	3	15	
	Motor movement changes	12	26	

a *Chi-square test p-value*.

b *KPS, Karnofsky Performance Score: an assessment tool for functional impairment*.

**Figure 4:**
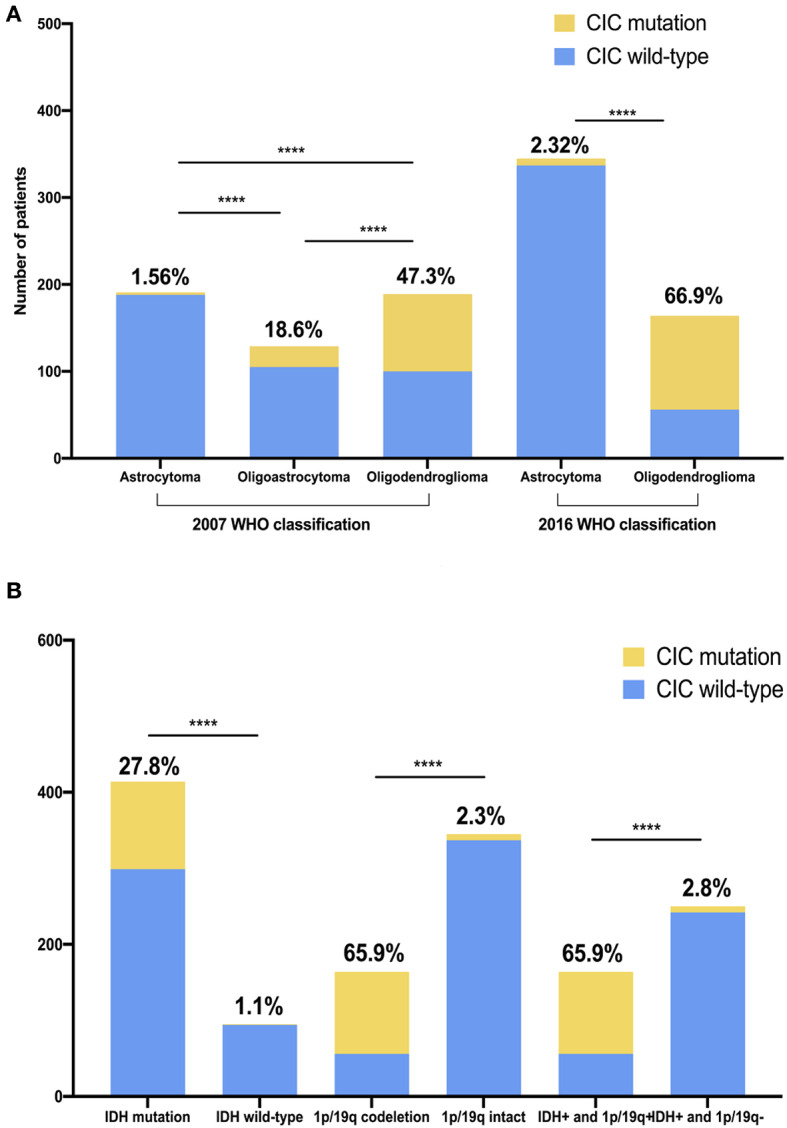
CIC mutation rate is unequal in different glioma classifications and genotypes. **(A)** CIC mutation in two different WHO CNS tumor classifications. **(B)** CIC mutation in different genotype glioma. IDH+, IDH mutation; 1p/19q+, 1p/19q codeletion; 1p/19q-, 1p/19q intact. The value above the bar is the ratio of CIC mutation. Chi-square test, ****P < 0.0001.

The TCGA classification for the glioma cohort is given using the 2007 WHO classification criteria. We reclassified all cases in our cohorts using the 2016 WHO classification criteria. CIC mutation was found in 65.9% of oligodendrogliomas and 2.32% of diffuse astrocytoma. The probability of CIC mutation occurring in oligodendroglioma is significantly greater than that in diffuse astrocytoma (*p* <1e−4) (Figure 4A, Table S3).

### Associations Between CIC Mutation and Genomic Data

We analyzed the association between CIC mutations and genomic data in the genomics dataset. CIC mutations are also closely related to some important molecular markers. IDH wild-type and CIC mutation is mutually exclusive. IDH mutation is found in nearly all patients with CIC mutation (99.1%) but only 1.1% IDH wild-type patients have CIC mutation. Similarly, almost all patients with CIC mutations have 1p/19q co-deletion (93.1%) but the proportion of CIC mutations in 1p/19q intact patients was only 2.32%, the probability of CIC mutations in 1p/19q co-deletion samples was significantly greater than 1p/19q intact samples (*p* < 1e−4). 78% of FUBP1 mutation patients had CIC mutation but the proportion of CIC mutations in FUBP1 wild-type patients was only 16.78%, the probability of CIC mutation in FUBP1 mutant samples is significantly greater than that of FUBP1 wild-type samples (*p* < 1e−4). Among the patients with CIC mutation, 93.1% had IDH mutation combined with 1p/19q co-deletion. 68.35% patients with IDH mutation combined with 1p/19q co-deletion had CIC mutation, but 2.28% other patients had CIC mutations. Patients with IDH mutation combined with 1p/19q co-deletion had a significantly higher probability of having CIC mutations than other samples (*p* < 1e−4) (Figure 4B, Table S3).

### Associations Between Overall Survival and CIC Mutation

**Figure 5:**
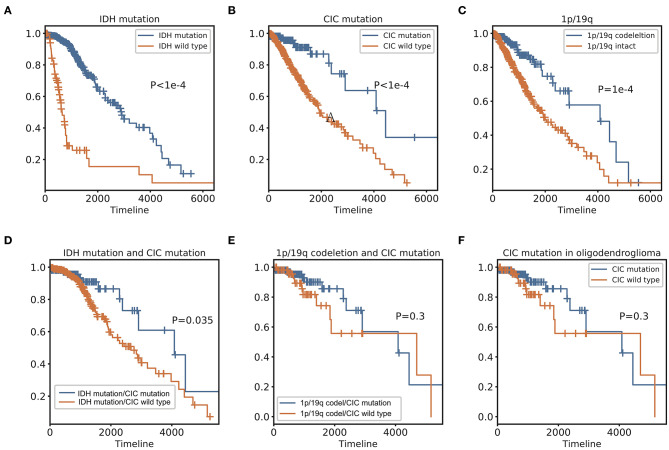
Kaplan Meier curve and Log-Rank test of IDH, 1p/19q, and CIC. **(A)** IDH mutation Kaplan-Meier curve shows that patients with IDH mutation have a significantly better prognosis than IDH wild type. **(B)** CIC mutation Kaplan-Meier curve shows that patients with CIC mutation have significantly longer OS than those whose CIC is wild type. **(C)** Patients with 1p/19q codeletion have significantly longer OS than those without 1p/19q codeletion. **(D)** Patients with IDH and CIC mutation have longer OS than patients have IDH mutation only. **(E)** There is no significant difference in OS between CIC mutation and CIC wild-type of patients with 1p/19q codeletion. **(F)** 2016 classification Oligodendroglioma. In the Log-Rank test, there is no significant survival difference between patients with and without CIC mutation.

In genomic dataset, IDH mutation patients have longer overall survival (OS) than IDH wild-type patients (*p* < 1e−4, Median OS (days): 2,907 vs. 648) (Figure 5A), CIC mutation patients have longer OS than CIC wild-type patients (*p* < 1e−4, Median OS (days): 4,445 vs. 1,933) (Figure 5B), 1p/19q codeletion patients have longer OS than 1p/19q intact patients (*p* = 1e−4, Median OS (days): 4,084 vs. 2,000) (Figure 5C, Table 2). Patients with IDH mutation combined CIC mutation have longer OS than those with IDH mutation only (*p* = 0.035, Median OS (days): 4,084 vs. 2,660) (Figure 5D), and there is also no significant difference between 1p/19q co-deletion patients with and without CIC mutation in our study (*p* = 0.3) (Figure 5E). Multivariate cox analysis including age, gender, grade, histological type, IDH mutation, 1p/19q codeletion and CIC mutation showed that IDH mutation and CIC mutation are both associated with better prognosis (*p* < 1e−4, HR = 0.3173; *p* < 1e−4, HR = 0.2445), and 1p/19q codeletion is not an independent prognostic factor (*p* = 0.3246, HR = 0.3322). Patients with IDH mutation combined 1p/19q codeletion don't have significant differences in OS with those with IDH mutation only (*p* = 0.1646, Median OS: 4,084 vs. 2,660) (Table 2). In log-rank test, CIC mutation doesn't show a significant association with OS in oligodendroglioma (*p* = 0.2992) (Figure 5F), but the multivariate cox analysis shows CIC mutation improves survival (*p* = 0.0362, HR = 0.3674) (Table 3).

**Table 2:** Survival analysis results.

	**Status**	**OS (median)^a^**	**Patients**	**Logrank_p^b^**	**Cox_p^c^**	**HR^d^**
IDH	Mutant	2,907	414	<1e-4	<1e-4	0.3173
	Wild-type	648	95			
CIC	Mutant	4,445	116	<1e-4	<1e-4	0.2445
	Wild-type	1,933	393			
1p/19q	Codeletion	4,084	164	1e-4	0.3246	0.3322
	Intact	2,000	345			
IDH mutant	1p/19q codeletion	4,084	164	0.1646	0.7011	0.8639
	1p/19q intact	2,660	345			
IDH mutant	CIC mutant	4,084	116	0.035	0.0287	0.4178
	CIC wild-type	2,660	393			

a *OS (median), the median overall survival time of the Kaplan Meier curve*.

b *Logrank_p, p-value of Log-Rank test*.

c *Cox_p, p-value of the Multivariate Cox test, including age, gender, grade, histological type, IDH mutation, 1p/19q codeletion, CIC mutation*.

d *HR, hazard ratio from the Multivariate Cox test*.

**Table 3:** Associations between CIC mutation and OS of patients with Oligodendroglioma.

**Classification**	**Subgroup**	**OS (median)**	**Logrank_p^b^**	**Cox_p^c^**	**HR^d^**
Oligodendroglioma^a^	CIC mutant	4,695	0.2992	0.0362	0.3674
	CIC wild-type				

a *Oligodendroglioma, 2016 WHO CNS tumor classification*.

b *Logrank_p, p-value of Log-Rank test*.

c *Cox_p, p-value of the Multivariate Cox test, including age, gender, grade, histological type, IDH mutation, 1p/19q codeletion, CIC mutation e*.

d *HR, hazard ratio from the Multivariate Cox test*.

### Image Feature Extraction and CIC Mutation Prediction

**Figure 6:**
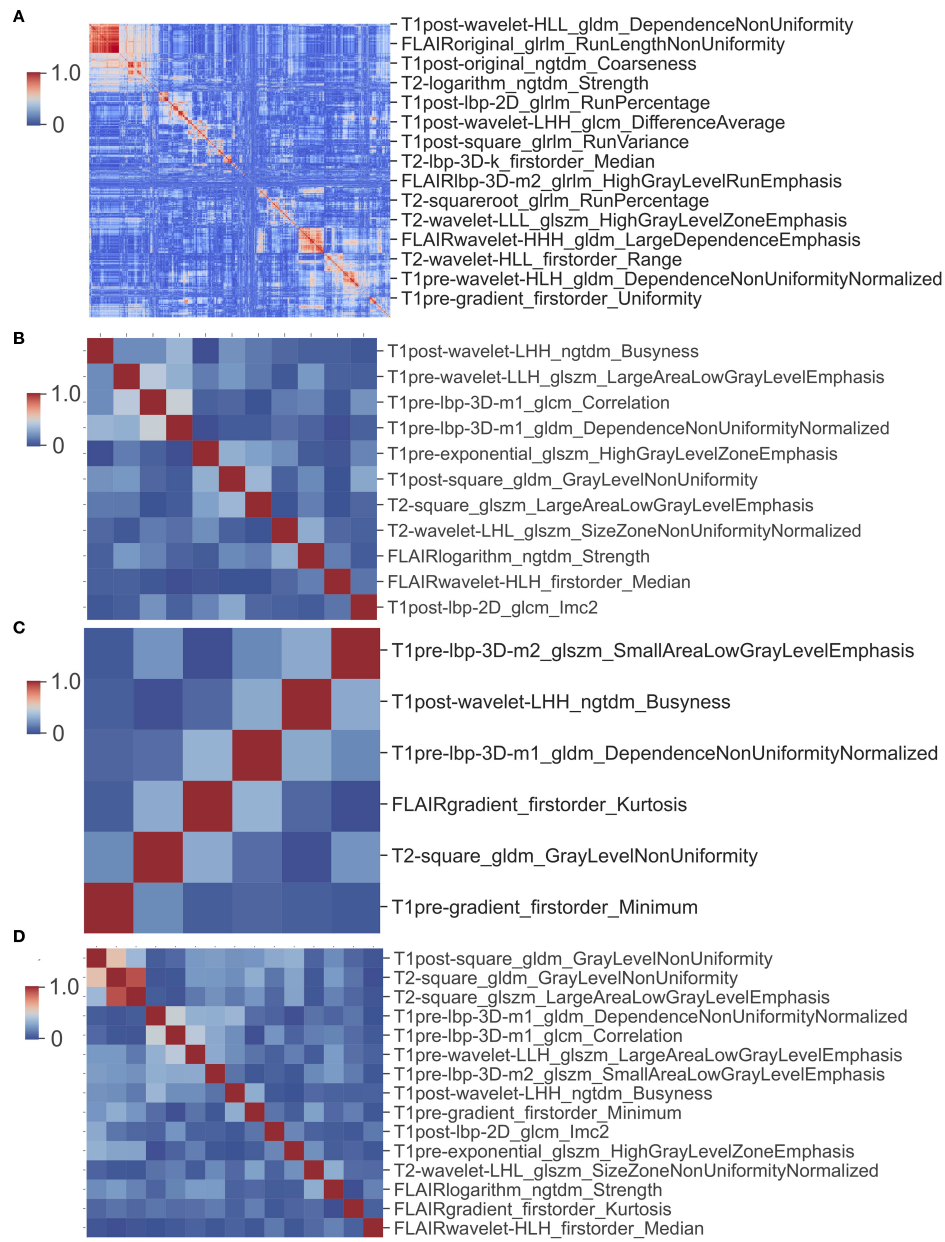
Cluster map of correlation between image features. **(A)** Cluster map of correlation of 6,676 image features. **(B)** Cluster map of correlation of 11 features used in glioma CIC mutation prediction model. **(C)** Cluster map of correlation of six features used in oligodendroglioma CIC mutation prediction model. **(D)** Cluster map of correlation between 15 image features from **(B)** and **(C)**.

A total of 1,669 features were extracted from each image (Table S1) and a total of 6,676 imaging features from T1W, T1CE, T2W, and FLAIR for each patient. The cluster map of the correlation of 6,676 features shows that there is collinearity between these features, but the collinearity is not very strong. Features can be clustered into some modules, but the size of modules are relatively small (Figure 6A). There is only one large module in the top-left (Figure 6A), but none of features in this module were selected to build the model.

**Figure 7:**
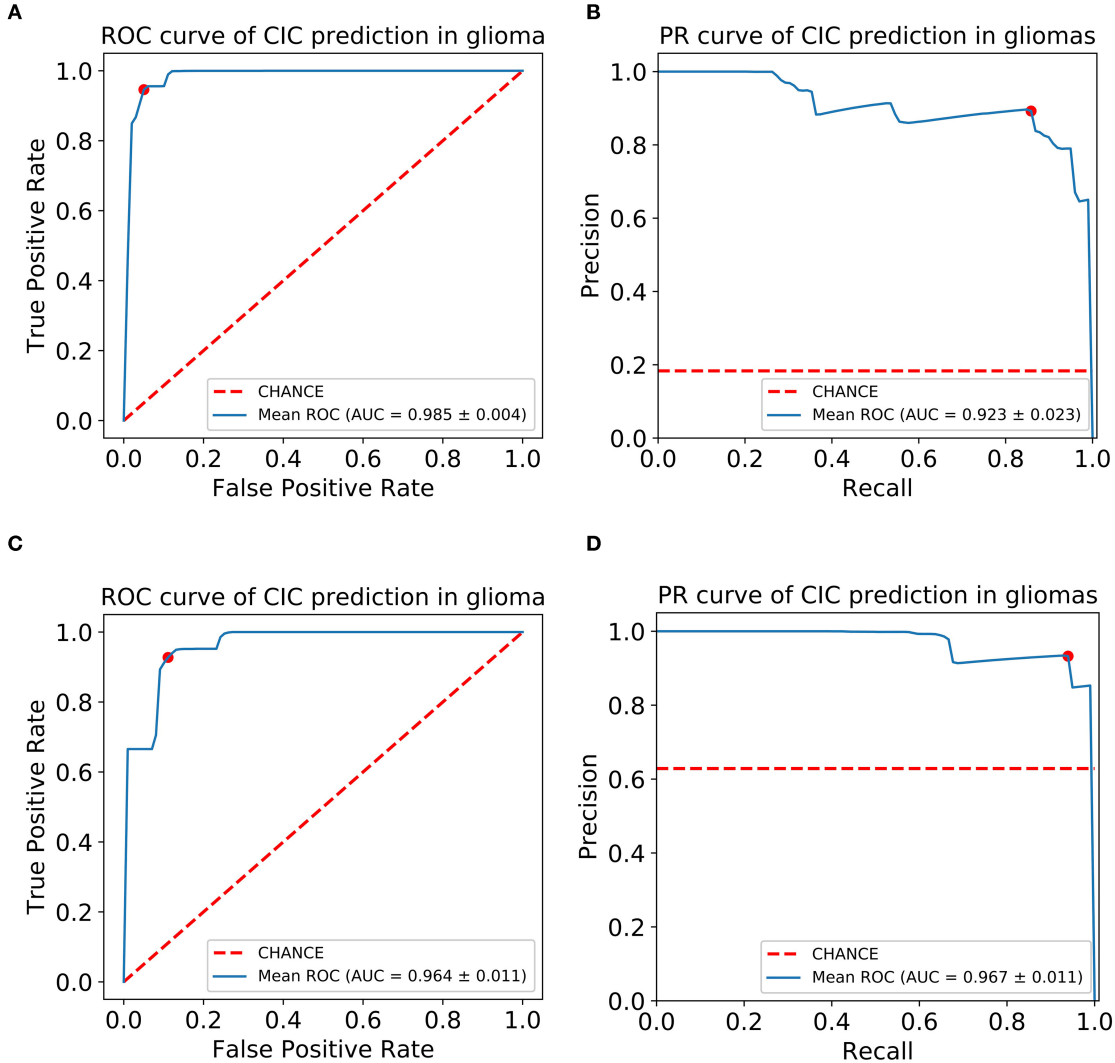
CIC mutation has a potential diagnosis value in glioma. **(A)** Receiver operating characteristic (ROC) curve of glioma CIC mutation prediction model. **(B)** Precision-recall (PR) curve of glioma CIC mutation prediction model. **(C)** Receiver operating characteristic (ROC) curve of oligodendroglioma CIC mutation prediction model. **(D)** Precision-recall (PR) curve of oligodendroglioma CIC mutation prediction model. The optimal cutoff value in ROC curve is the point (red dot) that has the smallest distance to (0,1), or (1,1) in PR curve.

For the prediction of CIC mutation in glioma, a total of 11 features were selected via Lasso regularization to build a logistic regression model (Table 4). The cluster map of the correlation of 11 features shows that the collinearity between features is weak (Figure 6B). The mean accuracy of the 1,000 repetition data split was 94.2% (95% CI, 94.03–94.38%), significantly higher than the no-information rate (81.7%). The mean AUC of the ROC curve was 0.985 (95% CI, 0.9841–0.9857) (Figure 7). The optimal cutoff value (0.0606) of the ROC curve exhibited a sensitivity, specificity, and accuracy of 94.83, 93.94, and 94.10%, respectively. The mean AUC of the Precision Recall (PR) curve is 0.923 (95% CI, 0.9183–0.9275). The optimal cutoff value (0.8485) of the PR curve exhibited sensitivity, precision, and accuracy of 84.85, 89.51, and 95.4%, respectively.

**Table 4:** Eleven features used to predict CIC mutation in gliomas.

**No**.	**Features**	**Importance^a^**	***P*-value^b^**
1	T2-wavelet-LHL_glszm_SizeZoneNonUniformityNormalized	2011.95	<10e-4
2	T1post-wavelet-LHH_ngtdm_Busyness	1842.85	0.0047
3	T1post-square_gldm_GrayLevelNonUniformity	1505.67	0.1402
4	FLAIR-wavelet-HLH_firstorder_Median	1046.31	0.0162
5	T2-square_glszm_LargeAreaLowGrayLevelEmphasis	1029.85	0.2475
6	T1pre-lbp-3D-m1_glcm_Correlation	935.72	0.0006
7	FLAIR-logarithm_ngtdm_Strength	758.25	0.5041
8	T1pre-wavelet-LLH_glszm_LargeAreaLowGrayLevelEmphasis	407.44	0.4334
9	T1post-lbp-2D_glcm_Imc2	−374.89	0.0013
10	T1pre-exponential_glszm_HighGrayLevelZoneEmphasis	270.67	0.0313
11	T1pre-lbp-3D-m1_gldm_DependenceNonUniformityNormalized	203.2	0.0009

a *Importance, Sum of coefficients of features in 1,000 prediction models*.

b *P-value: Mann-Whitney U-test p-value*.

For the prediction of CIC mutation in Oligodendroglioma, a total of six features from 35 Oligodendroglioma patients were selected via Lasso regularization to build a logistic regression model (Table 5). The cluster map of the correlation of six features shows that the collinearity between features is weak (Figure 6C). There are two features that overlap with the 11 features set above. The cluster map of the correlation of 15 features shows that the collinearity between features is weak except T2-square_glszm_LargeAreaLowGrayLevelEmphasis and T2-square_gldm_GrayLevelNonUniformity (Figure 6D). The mean accuracy of the 1,000 repetition data split was 92.3% (95% CI, 91.70–92.92%), significantly higher than the no-information rate (62.9%). The mean AUC of the ROC curve is 0.967 (95% CI, 0.9643–0.9687) (Figure 7). The optimal cutoff value (0.1010) of the ROC curve exhibited a sensitivity, specificity, and accuracy of 94.26, 89.90, and 92.64%, respectively. The mean AUC of the PR curve is 0.9705 (95% CI, 0.9684–0.9726). The optimal cutoff value (0.9596) of the PR curve exhibited sensitivity, precision, and accuracy of 95.96, 93.51, and 93.27%, respectively.

**Table 5:** Six features used to predict CIC mutation in oligodendrogliomas.

**No**.	**Features**	**Importance^a^**	***P*-value^b^**
1	T1pre-lbp-3D-m2_glszm_SmallAreaLowGrayLevelEmphasis	2022.39	0.2209
2	T1post-wavelet-LHH_ngtdm_Busyness	1527.58	0.0047
3	T2-square_gldm_GrayLevelNonUniformity	1140.3	0.2108
4	T1pre-lbp-3D-m1_gldm_DependenceNonUniformityNormalized	696.76	0.0009
5	T1pre-gradient_firstorder_Minimum	−694.92	0.6766
6	FLAIR-gradient_firstorder_Kurtosis	81.75	0.0096

a *Importance, Sum of coefficients of features in 1,000 prediction models*.

b *P-value, Mann-Whitney U-test p-value*.

### Image Feature Analysis

**Figure 8:**
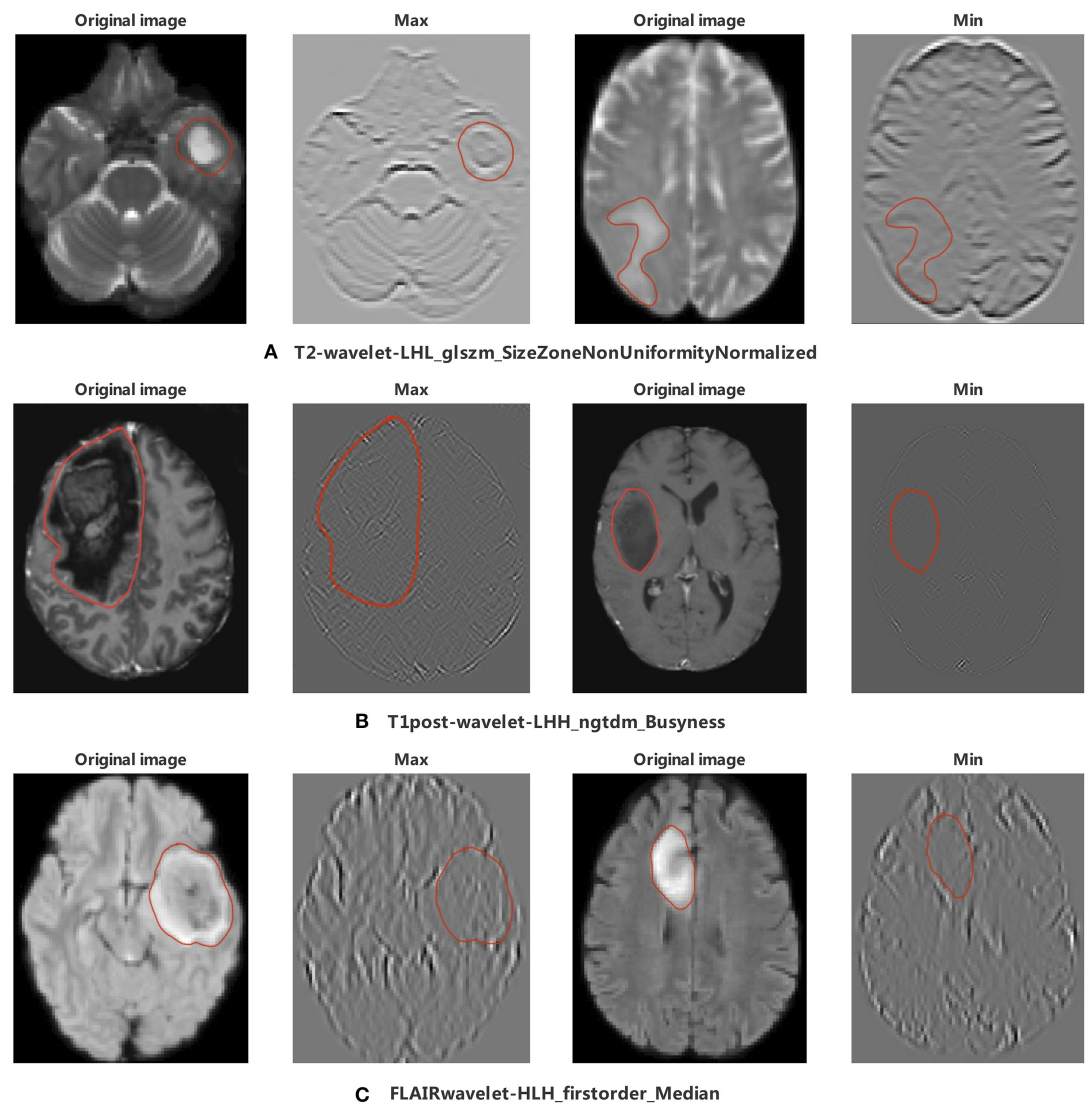
The appearance of the MR image corresponding to the top 3 radiomic features in glioma. The region encircled in red represents the lesion boundary. **(A)** Images corresponding to the maximum and minimum of T2-wavelet-LHL_glszm_SizeZoneNonUniformityNormalized. **(B)** First column images are the Wavelet images corresponding to the maximum and minimum value of T1post-wavelet_LHH_ngtdm_Busyness, second column images are original images of wavelet images. **(C)** Images corresponding to the maximum and minimum value of FLAIR-wavelet-HLH_firstorder_Median.

**Figure 9:**
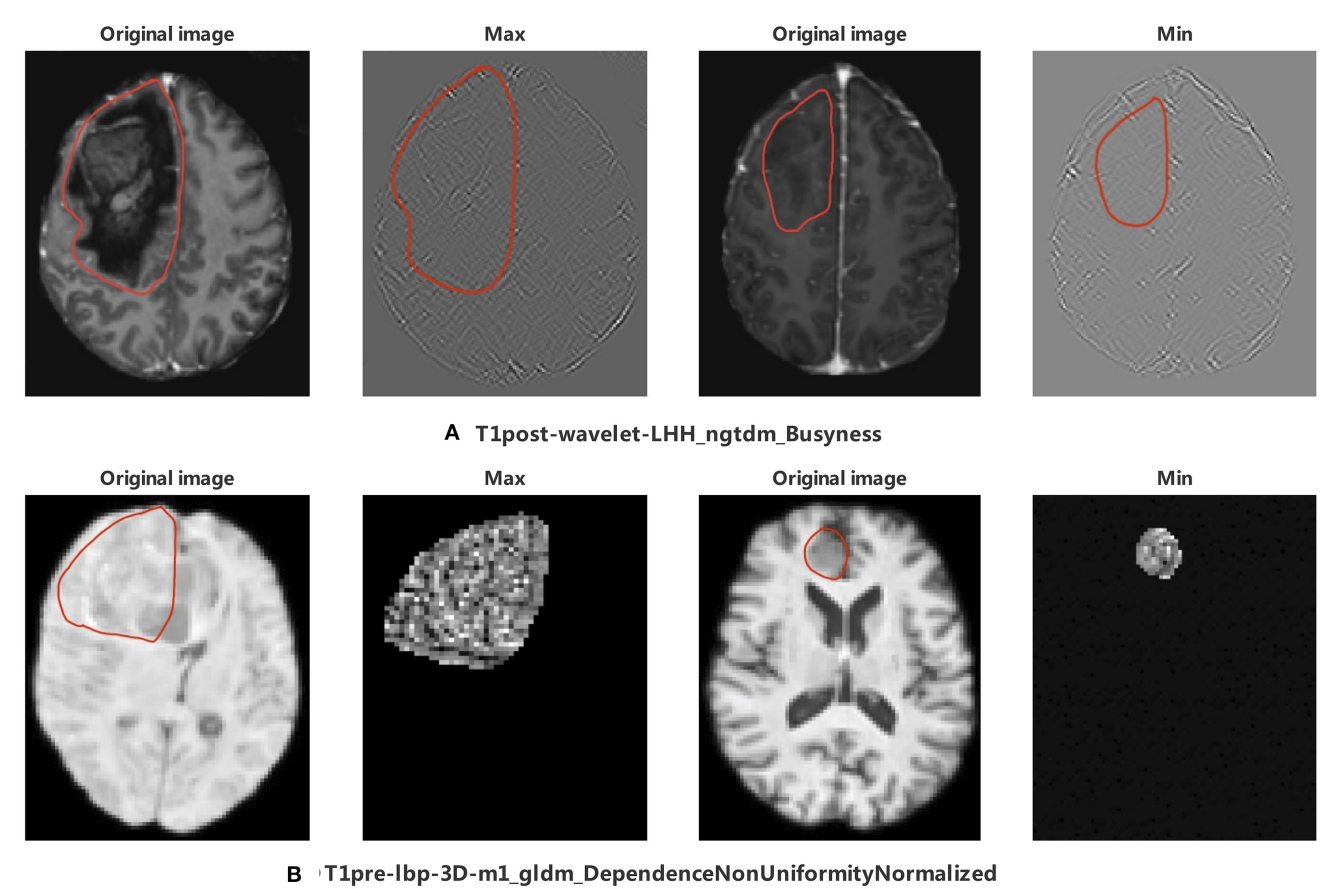
The appearance of the MR image corresponds to top 2 radiomics features in Oligodendroglioma. The region encircled in red represents the legion boundary. **(A)** wavelet images and corresponding original images of the maximum and minimum value of T1post-wavelet_LHH_ngtdm_Busyness. **(B)** LBP images and corresponding original images of the maximum and minimum value of T1pre-lbp-3D-m1_gldm_DependenceNonUniformityNormalized. LBP image only included mask region in PyRadiomics (35).

**Figure 10:**
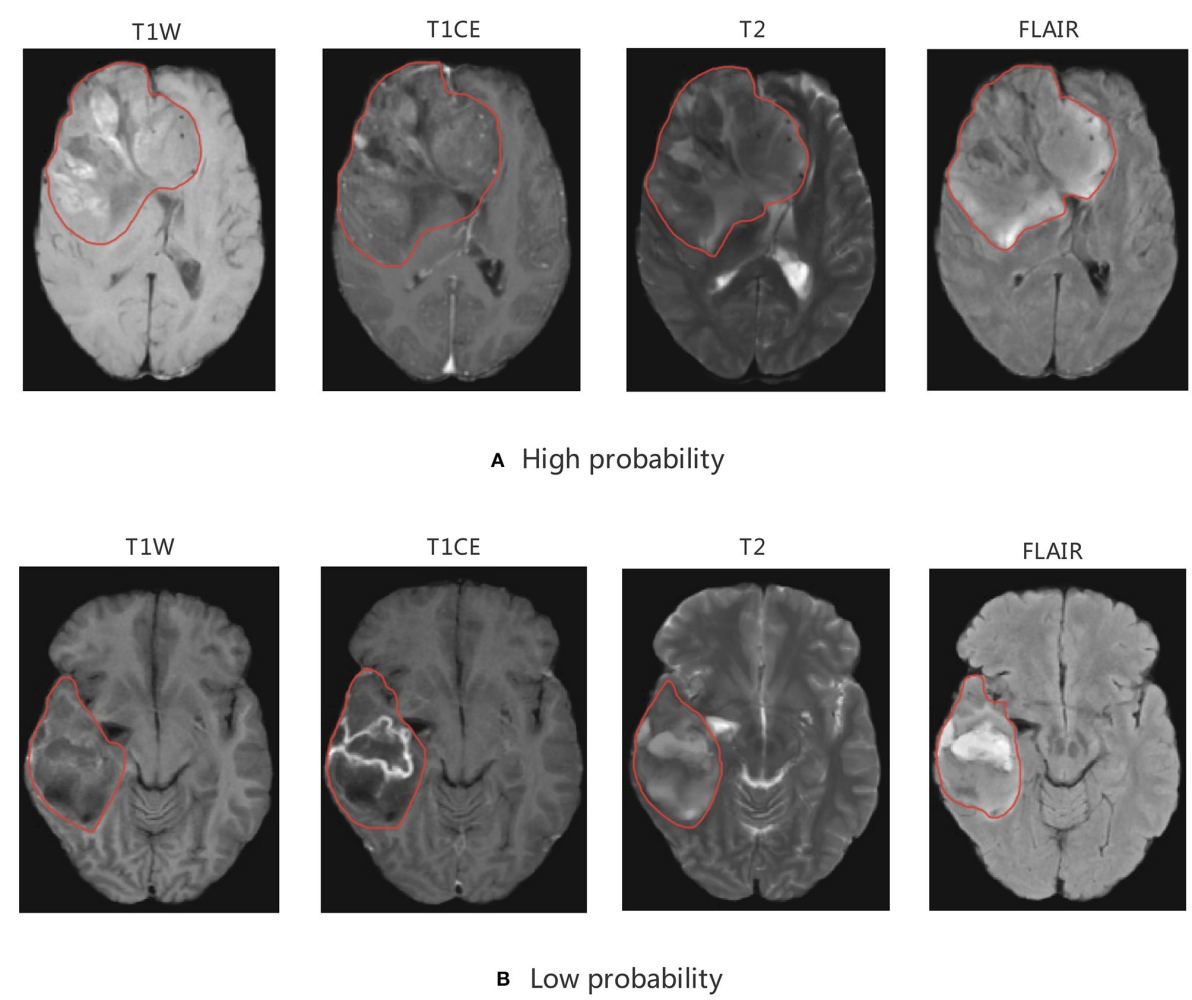
MRI of model-based CIC mutation prediction patients. Image with the **(A)** high probability of being CIC mutation and **(B)** low probability of being CIC mutation. The region inside the red line represents glioma region.

Among the 11 features of the logistic regression model of CIC mutation prediction in gliomas, seven features were found significant (Mann-Whitney *U*-test, alpha = 0.05). To help illustrate some of these imaging characteristics, we extracted 2D image slices that maximize or minimize the top 3 selected features (Figure 8). T2-wavelet-LHL_glszm_SizeZoneNonUniformityNormalized, T1post-wavelet-LHH_ngtdm_Busyness, and FLAIR-wavelet-HLH_firstorder_Median are the top 3 significant features. Among the six features of the logistic regression model of CIC mutation prediction in Oligodendrogliomas (Table S4, Figure 9), three features were found to be significant (Mann-Whitney *U*-test, alpha = 0.05). T1post-wavelet-LHH_ngtdm_Busyness and T1pre-lbp-3D-m1_gldm_DependenceNonUniformityNormalized were the top 2 significant features. Images corresponding to the highest and lowest probability of CIC mutation (based on logistic regression) were selected (Figure 10).

## Discussion

In our study, we utilized TCGA and TCIA to explore the potential to predict genomics based on MR images. We found that CIC mutation has excellent diagnostic value in LGG, and that CIC mutation is mutually exclusive with glioblastoma (Figure 3), so we excluded the TCGA GBM cohort from further study. CIC mutation mainly occurs in IDH mutation and 1p/19q co-deletion patients and is mutually exclusive with IDH wild-type or 1p/19q intact patients (Figure 4). CIC mutation is also associated with clinical characteristics as described in (Table 1). We can speculate IDH mutation, 1p/19q codeletion, and histological type information from CIC mutation status. Grade 2 patients and elder patients are more likely to have CIC mutation. Interestingly, patients without clinical symptoms (headache, visual changes, sensory changes) have higher probability of having CIC mutation. This is probably seen because CIC mutations occur primarily in grade 2 gliomas and oligodendrogliomas. These grades of gliomas have a lower degree of malignancy, slower disease progression, and clinical symptoms occur later and are not obvious, leading to patients being diagnosed at an older age.

In our study, CIC mutation is present in 65.9% of 1p/19q codeletion patients, but rarely in 1p/19q intact patients (2.3%). In order to find out whether the significant correlation between CIC mutations and OS comes from the 1p/19q codeletion, we performed multivariate cox regression analysis. In the result of multivariate cox regression analysis, which included IDH mutation, 1p/19q codeletion, CIC mutation, age, gender, grade, and histological type, CIC mutation is significant (*p* < 1e−4), but 1p/19q codeletion is not significant (*p* = 0.3246) (Table 2). We also found that CIC mutation in IDH mutation patients is associated with a better prognosis (*p* = 0.0287, HR = 0.4178), but there is no significant difference between 1p/19q codeletion and prognosis (*p* = 0.7011) (Table 2). In addition, in oligodendroglioma (IDH mutation and 1p/19q codeletion) patients, CIC mutation is associated with a better prognosis (*p* = 0.0362). It was reported that almost every glioma with a CIC or FUBP1 mutation exhibited an IDH gene mutation (13). Thus, we also analyzed FUBP1 status in our study. There are 50 FUBP1 mutation patients, almost all FUBP1 patients exhibited an IDH mutation, and most FUBP1 mutation patients combined CIC mutation. But not all CIC mutation patients have FUBP1 mutation. We did multivariate cox analysis including FUBP1 mutation and found no significant association between FUBP1 mutation and survival (*p* = 0.2959) (Table S4). Therefore, CIC mutation was an independent good prognostic factor in our study.

As reported, gliomas with different genotypes have different MRI appearances (43–46). In our study we focused on 11 extracted radiomics features. The top 2 of these 11 radiomic features were T2-wavelet-LHL _glszm_SizeZoneNonUniformityNormalized (SZNN) and T1post-wavelet-LHH_ngtdm_Busyness (Busyness). Both features are extracted from wavelet transform images which reflect fine details of the original images. SZNN measures the variability of size zone volumes throughout the image, with a lower value indicating more homogeneity among zone size volumes in the image (35). Busyness is a measure of the change from a pixel to its neighbor. A high Busyness indicates rapid changes in intensity (35). In other words, both Busyness and SZNN are measures of image heterogeneity and non-uniformity. SZNN minimal value corresponding image is flat but the tumor region appears as obvious protruding ridges and depressed trenches in the maximum value (Figure 8).

CIC mutation mainly occurs in Oligodendroglioma but not all. To identify the difference between Oligodendrogliomas with and without CIC mutations, six features were selected after the Lasso process. Top 2 features are T1post-wavelet-LHH_ngtdm_Busyness (Busyness) and T1pre-lbp-3D-m1_gldm_ DependenceNonUniformityNormalized (DNN). DNN measures the similarity of dependence defined as the number of connected voxels within distance δ that are dependent on the center voxel, a lower value indicating more homogeneity among dependencies in the image (35) (Figure 9). The DNN max value corresponding image is extremely complex and heterogeneous but the tumor region appears smaller and simpler in the minimum value. From the images, we find that the appearance of Oligodendrogliomas with or without CIC mutation are similar, not as obvious as the difference between all types of gliomas with or without CIC mutation. Both Oligodendrogliomas with or without CIC mutation are heterogeneous and non-uniform. But according to the meaning of image features, we still speculate that Oligodendroglioma with CIC mutation still appears more heterogeneous and complex.

As discussed above, CIC mutation suggests a better prognosis in patients with IDH mutation and 1p/19q codeletion. Therefore, we concluded that patients with CIC mutation have the best prognosis and longest survival. In our study, most CIC mutation gliomas have a relatively larger tumor region, more obvious mass effect, greater non-uniformity, heterogeneity, and scattered areas of intratumorally necrosis with or without corresponding areas of contrast enhancement. The special appearance may be due to the low malignancy of glioma with CIC mutation. The relatively weak proliferative, invasive and migration ability leads to CIC mutation gliomas growing slowly, resulting in not obvious clinical symptoms and larger tumor volume. This is consistent with published results that show CIC mutation is more likely to occur in patients of older age, grade 2 glioma, and without clinical symptoms. The cause for scattered areas of weak contrast enhancement may be that the tumor is less malignant, resulting in slow tumor growth, less ischemia and hypoxia, and less damage to the blood brain barrier (BBB) which prevents media from leaking through the BBB. On the other hand, gliomas which have a small tumor region but severe necrosis, obvious contrast enhancement, and obvious peritumoral edema (reflects rapid growth) which indicated strong invasion and severe BBB damage have a lower probability of CIC mutation.

Although radiomic features perform well, there are some limitations to our study analysis. First, all data is from public datasets (TCGA and TCIA), which displays large variance in quality of images that may influence predictive analysis. Second, data was imbalanced because of the low incidence of CIC mutation. Third, only structural MRIs were included. Functional and diffusion-weighted MR images are an area of interest that could be included in similar analysis in future work. Lastly, in this study, all images were obtained from one cohort (TCIA). Future work could benefit from using a second independent cohort for testing, which would provide a better measure of model generalizability / reliability.

In conclusion, our results support CIC mutation status as a valuable diagnostic and prognostic biomarker of lower-grade glioma. We showed that CIC mutation could be accurately predicted by MRI radiomic features. MRI of CIC mutation gliomas were found to display visually less malignant manifestations, such as milder necrosis and larger tumor volume. Radiomics plays an important role in the accurate diagnosis and personalized treatment of gliomas. The exploration of its association with medical imaging appearance and its clinical application are worth further efforts.

## Data Availability Statement

Publicly available datasets were analyzed in this study. This data can be found here: https://portal.gdc.cancer.gov/, https://www.cancerimagingarchive.net/.

## Author Contributions

LZ and FG contributed to experimental design, data analysis, and manuscript writing. DG, XL, and JV contributed to experimental design and manuscript writing. All authors contributed to the article and approved the submitted version.

## Conflict of Interest

The authors declare that the research was conducted in the absence of any commercial or financial relationships that could be construed as a potential conflict of interest.
