# Supplementary material for: Radiomics Features Predict CIC Mutation Status in Lower Grade Glioma
Source: Front Oncol. 2020 Jun 26;10:937. doi: 10.3389/fonc.2020.00937 (PMC7333647; doi:10.3389/fonc.2020.00937)
Supplement: Supplementary file 1 [file Data_Sheet_1.DOCX]

Supplementary Material

# Supplementary Figures and Tables

## Supplementary Tables

**Table S1** List of radiomic features extracted for features selection.

| **Feature category** | **Feature list** |
| --- | --- |
| First order (n=19) | Energy, Total Energy, Entropy, Minimum, 10th percentile, 90th percentile, Maximum, Mean, Median, Interquartile Range, Range, Mean Absolute Deviation, Robust Mean Absolute Deviation, Root Mean Squared, Standard Deviation, Skewness, Kurtosis, Variance, Uniformity |
|  |  |
|  |  |
| Shape (n=16) | Volume, Surface Area, Surface Area to Volume ratio, Sphericity, Compactness 1, Compactness 2, Spherical Disproportion, Maximum 3D diameter, Maximum 2D diameter(Slice), Maximum 2D diameter(Column), Maximum 2D diameter(Row), Major Axis Length, Minor Axis Length, Least Axis Length, Elongation, Flatness |
|  |  |
| GLCM^1^ (n=24) | Autocorrelation, Joint Average, Cluster Prominence, Cluster Shade, Cluster Tendency, Contrast, Correlation, Difference Average, Difference Entropy, Difference Variance, Joint Energy, Joint entropy, Informal Measure of Correlation 1, Informal Measure of Correlation 2, Inverse Difference Moment,  Inverse Difference Moment Normalized, Inverse Difference, Inverse Difference Normalized, Inverse Variance, Maximum Probability, Sum Average, Sum Entropy, Sum of Squares |
|  |  |
| GLSZM^2^ (n=16) | Small Area Emphasis, Large Area Emphasis, Gray Level Non-Uniformity, Gray Level Non-Uniformity Normalized, Size-Zone Non-Uniformity, Size-Zone Non-Uniformity Normalized, Zone Percentage, Gray Level Variance, Zone Variance, Zone Entropy, Low Gray Level Zone Emphasis, High Gray Level Zone Emphasis, Small Area Low Gray-Level Emphasis, Small Area High Gray-Level Emphasis, Large Area Low Gray-Level Emphasis, Large Area High Gray-Level Emphasis |
|  |  |
| GLRLM^3^ (n=16) | Short Run Emphasis, Long Run Emphasis, Gray Level Non-Uniformity, Gray Level Non-Uniformity Normalized, Run Length Non-Uniformity, Run Length Non-Uniformity Normalized, Run Percentage, Gray Level Variance, Run Variance, Run Entropy, Low Gray Level Run Emphasis, High Gray Level Run Emphasis, Short Run Low Gray-Level Emphasis, Short Run High Gray-Level Emphasis, Long Run Low Gray-Level Emphasis, Long Run High Gray-Level Emphasis |
|  |  |
| NGTDM^4^ (n=5) | Coarseness, Contrast, Busyness, Complexity, Strength |
|  |  |
| GLDM^5^ (n=14) | Small Dependence Emphasis, Large Dependence Emphasis, Gray Level Non-Uniformity, Dependence Non-Uniformity, Dependence Non-Uniformity Normalized, Gray Level Variance, Dependence Variance, Dependence Entropy, Low Gray-Level Emphasis, High Gray-Level Emphasis, Small Dependence Low Gray-Level Emphasis, Small Dependence High   Emphasis, Large Dependence Low Gray-Level Emphasis, Large Dependence High Gray-Level Emphasis |

GLCM^1^: Gray Level Co-occurrence Matrix; GLSZM^2^: Gray Level Size Zone Matrix; GLRLM^3^: Gray Level Run Length Matrix; NGTDM^4^: Neighboring Gray Tone Difference Matrix; GLDM^5^: Gray Level Dependence Matrix.

**Table S2** Clinical and genomic characteristics of the genomic dataset and image dataset.

| Characteristics | Subgroup | Genomic dataset (n=509) | Image dataset (n=120) | P value^1^ |
| --- | --- | --- | --- | --- |
| Mean overall survival |  | 955 | 959 | 0.9698 |
| Number of deaths observed |  | 126 (24.8%) | 33 (27.5%) | 0.5336 |
| KPS^2^ |  | 87.4 | 86.7 | 0.6375 |
| Mean Age(year) |  | 43 | 45.9 | 0.0356 |
| Gender | FEMALE | 227 | 63 | 0.1182 |
|  | MALE | 282 | 57 |  |
| grade | G2 | 247 | 58 | 0.9696 |
|  | G3 | 262 | 62 |  |
| Cancer status | WITH TUMOR | 269 | 74 | 0.0858 |
|  | TUMOR FREE | 178 | 33 |  |
| tumor location | Frontal Lobe | 298 | 69 | 0.6876 |
|  | Temporal Lobe | 145 | 33 |  |
|  | Parietal Lobe | 46 | 14 |  |
| IDH | mutant | 414 | 96 | 0.7368 |
|  | wild-type | 95 | 24 |  |
| 1p/19q | codeletion | 164 | 35 | 0.5176 |
|  | intact | 345 | 85 |  |
| CIC | mutant | 116 | 22 | 0.2886 |
|  | wild-type | 393 | 98 |  |
| 2007 Classification^6^ | A^3^ | 192 | 35 | 0.1863 |
|  | OA^4^ | 129 | 32 |  |
|  | O^5^ | 188 | 53 |  |
| 2016 Classification^7^ | A | 345 | 85 | 0.5176 |
|  | O | 164 | 35 |  |

^1^P value: Chi-square test, ^2^KPS: Karnofsky performance score. ^3^A: Astrocytoma; ^4^OA: Oligoastrocytoma; ^5^O: Oligodendroglioma. ^6^2007 classification: 2007 WHO CNS tumor classification, classify gliomas based on microscopic similarities with different putative cells of origin; ^7^2016 Classification: 2016 WHO CNS tumor classification, integrated phenotypic and molecular biomarker, IDH mutation and 1p/19q codeletion.

**Table S3** CIC mutation status in different classifications and molecular alterations.

| Variables | subgroup | CIC mutant | CIC wild-type | P value |
| --- | --- | --- | --- | --- |
| 2007 classification | A | 3 | 189 | <10e-4 |
|  | OA | 24 | 105 |  |
|  | O | 89 | 99 | <10e-4 |
|  | A | 3 | 189 |  |
|  | OA | 24 | 105 | <10e-4 |
|  | O | 89 | 99 |  |
| 2016 classification | A | 8 | 337 | <10e-4 |
|  | O | 108 | 56 |  |
| IDH mutant | YES | 115 | 299 | <10e-4 |
|  | NO | 1 | 94 |  |
| 1p/19q codeletion | YES | 108 | 56 | <10e-4 |
|  | NO | 8 | 337 |  |
| IDH mutant+1p/19q codeletion | YES | 108 | 50 | <10e-4 |
|  | NO | 8 | 343 |  |

P-value: p-value of Chi-Square test; A: Astrocytoma; OA: Oligoastrocytoma; O: Oligodendroglioma.

| **Table S4** (A) FUBP1 mutation distribution | | | | |
| --- | --- | --- | --- | --- |
|  | CIC mutation | CIC wild-type | IDH mutation | IDH wild-type |
| FUBP1 mutation | 39 | 11 | 49 | 1 |
| FUBP1 wild-type | 77 | 382 | 365 | 94 |
|  |  |  |  |  |
| (B) Multivariate cox regression of FUBP1 mutation | | | | |
|  | HR | HR lower 95% | HR upper 95% | p |
| CIC | 0.2752 | 0.1321 | 0.5733 | 0.0006 |
| Age | 1.0503 | 1.0336 | 1.0672 | 1.91E-09 |
| Grade | 2.6022 | 1.7022 | 3.9782 | 1.01E-05 |
| Histology | 1.1876 | 0.9234 | 1.5274 | 0.1806 |
| Gender | 0.9797 | 0.6797 | 1.4121 | 0.9125 |
| IDH | 0.3077 | 0.1934 | 0.4896 | 6.6E-07 |
| 1p19q | 1.5026 | 0.7632 | 2.9584 | 0.2388 |
| FUBP1 | 0.6267 | 0.2609 | 1.5052 | 0.2959 |

## Supplementary Figures

##
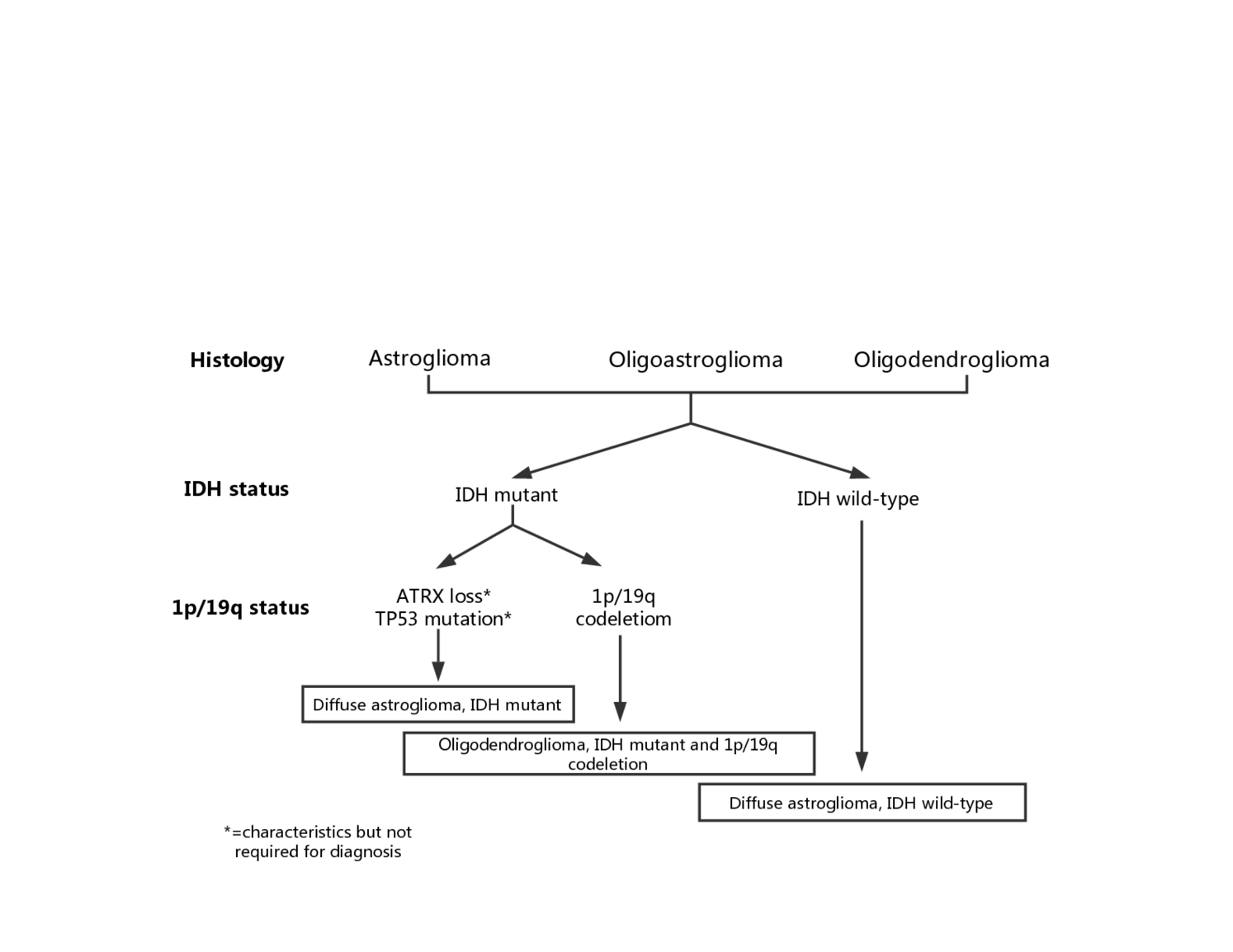


**Supplementary Figure 1.** A simplified algorithm for classification of the diffuse gliomas based on histological and genetic features.

**Supplementary Figure 2.** Radiomics features selection

**Supplementary Figure 3.** Iteration of LassoCV

**Supplementary Figure 4.** CIC mutation prediction validation.

**Supplementary Figure 5.** CIC mutation in GBM and LGG.
